# A New Method of Treating Carbuncle

**Published:** 1896-12

**Authors:** T. C. Osborn

**Affiliations:** Cleburne, Texas


For the Texas Medical Journal.

### A NEW METHOD OF TREATING CARBUNCLE.

BY T. C. OSBORN, M. D., CLEBURNE, TEXAS.

[Read before the Johnson County Medical Society on the 1st of October, 1896.]

IN MAY, 1894, I had the honor of presenting a paper to this society on the abortion of small-pox by the external application of a ten per cent. solution of bichloride of mercury, *in water*, twice over the whole body. At that time I conscientiously believed that I was pioneering a new and rational method of treating variola, and fervently predicted that sooner or later the ideas then advanced would be recognized by the profession. My hope has become realized as far as the success of the treatment was concerned, but my priority is disputed by the pretensions of three or more claimants within the last few months. These pretensions do not vex me. On the contrary, it is very gratifying to m y feelings to be assured in this way that the adoption of the practice will not only result in the saving of thousands of lives by its safety and simplicity, but also that it will forever dissipate all the horror of the contagiousness of the disease, prevent the pitting; and by the use of the sponging with the medicine on suspects, and their clothing, no danger of infection need be apprehended, nor dread of spreading in the community wherein one case has been unwittingly introduced. No, it need go no further. Then will come the millenium; its disappearance among men, and with it will come the end of quarantine camps, and State and municipal expenditures, and, finally, the extinction of vaccination, with all its dangers and uncertainties. The coming of such humane benefits may not be in my day; but no matter, as the living will be the recipients; it matters not who shall be entitled to the claim of priority in its introduction. Not that I have no pride in the matter. On the contrary, I am "still in the ring," as sportsmen say, because every claimant that I have so far seen has used the *alcoholic* solution, which, on account of its greater danger of toxic effects, is too hazardous, in my estimation, to come into general practice. Others, however, have used it and prize the success it has attained. I do not use the bichloride of mercury on the skin in alcohol, because of its bad behavior in patients of mine nearly fifty years ago. The watery solution is less dangerous, and that is the preparation having my confidence. With this provision I do still claim priority in the discovery.

To-night, gentlemen, it is my earnest desire to engage your attention while I disclose to you another "flash of light on my path," in relation to a discovery in practice, which, whilst in no wise as important as the jugulation of small-pox, is, nevertheless, sufficiently so to command a profound interest in its practical effects.

Carbuncle is, without doubt, an evidence of a serious form of protracted indigestion. It is highly inflammatory, and has been known to cause death. At all times it is an exceedingly painful disease, pursuing, under the old management, a progressive course until the involved cellular tissue has sloughed away. The high grade of fever accompanying it is conclusive proof of its distressing effects upon the nerves and circulation. German physicians teach that carbuncles constitute a critical evacuation of diseased humors, but this I do not believe; on the contrary, I am well satisfied that the "diseased humors," if any there are, have no other claim to consideration than is involved in the preceding or antecedent indigestion. And this belief caused the question in my mind as to the appropriateness of placing the treatment of it in the surgical repertory. My trust now is that in future it will be laid strictly in the medical department. Surgery is done with it; no knives, nor caustics any longer. If this were the only advantage of its adoption in practice, its benefits could not well be overestimated. No one will deny the assertion that the old management of carbuncles was unsatisfactory. It resolved itself into an expectant method. The cautery, and the scalpel, with anodynes to lessen its painful tension, made up the sole armamentarium for the encounter, under the flag of hope and patience, until the gangrenous slough should become detached from the base of the agonizing tumor.

After declaring to you that for more than a year it has been my fixed intention to disclose the secret of my success in the treatment of carbuncles, it is to be feared that my delay is open to censure, and the only excuse I can or will offer is the commendable fact that I was waiting for further observations in confirmation of my theory to eliminate all possible charges of coincidence in its discussion.

I trust it will not be out of place just here to mention a peculiarity which, according to my observations, should obtain considerable prominence in the clinical history of carbuncle. The disease, as recorded in my notes during a little over half of the present century, has seemed to be neither recurrent nor infectious. There is no record in any of the authorities going to show that it has passed by contact, or by atmospheric influence, from one person to another; nor has it ever recurred a second time in the same individual. It may, therefore, be classed, rather anomalously, as non-recurrent, and non-contagious. And yet, whilst I am astride the fence on the theories of bacilli, because I could never divest my mind of the vexatious doubt of which was *first*, the disease or the bacillus, it is, nevertheless, clear to me that anthrax, as a local affliction, must assuredly have a special germ for its production. Please notice that I have used the terms carbuncle, and anthrax synonymously, whilst aware, at the same time, that there has grown up a technical difference between them. If there is a difference, in my sight it is too microscopical to disturb the views of my college impressions.

The above remarks are introduced, not so much for their intrinsic worth, as for my desire to elicit your discussion on the subject. If I am wrong, I am also open to conviction, whenever sufficient evidence is brought to bear upon my judgment.

Having in this manner disposed of my theories in reference to carbuncles, I am all the more ready to unburden myself of the desire to tender to you the details of my late experience in aborting, in mid-career, the disease under consideration. And, that I may not be misunderstood in claiming priority, I will state that the reality of the secret consists in the happy selection of the Kumpe tonic as the prime agent by which the affliction was jugulated. This medicine is best known and most popular with the profession in Alabama, where the distinguished originator of it, Dr. George E. Kumpe, first divulged its formula in a paper on the fevers of the State, at the annual meeting of the Alabama State Medical Association in 1870. At that time the profession there was greatly annoyed at being unable to arrest and control the wide-spread relapses of intermittent fever; and gladly availed themselves of any proposed remedy coming from reliable sources.

Dr. Kumpe spoke of the tonic as being very efficient in his hands, and commended it in such terms as to preclude any doubt of its efficiency in arresting stubborn cases of intermittent fever, after the attack had been temporarily checked by the sulphate of quinine. He did not speak of it in any other connection. I, however, became so partial to it that, to this day, if a tonic influence is necessary in my clientelle, the "Kumpe" is almost always the agent which is the choice preferred. From this statement you will infer that the inspiration actuating my selection amongst the great number of its class was entirely accidental, and by no means reasoned out by any precepts of ratiocination. The fact is, I use it to the exclusion of all others, except one, and that exception is in favor the Tillbury Fox's tonic in the treatment of eczema. I have come to believe that all skin diseases are best treated by tonic medicines.

The formula for making the mixture is like this:

℞ Quinia sulphas .............................. grs. xxx

Tinct. ferri chloridi ........................ ʒjv

Liq. potas. arsenitis........................ ʒj

Strychnia sulphas .......................... gr. ss

Tinct opii ....................................... ʒj

Vin madeira ad .............................. ℥viij

Sig.: For adults, one table spoonful in ¼ glassful of water, three times a day, preferably just after meals.

But at the next meeting of the State Association, Dr. E. D. McDaniel, now professor of Materia Medica in the Mobile Medical College, suggested an amendment to the formula, making water instead of wine, the solvent in the combination, as under his administration of it the wine caused too much precipitation of its ingredients. This was concurred in by Dr. Kumpe and all the members who had, in the meantime, given the mixture sufficient trials to warrant a second discussion on its merits. I feel sure this explanation will be amply sufficient in my preference for the "Kumpe tonic," in all cases where tonic medicines are necessary in the treatment of any stated disease.

Owing to the rush of their preparations by the various pharmaceutical establishments, some of which have nearly the same ingredients as has the Kumpe, and far more elegant and concentrated in material and form, for which I am duly grateful, yet, notwithstanding all this, and the fact that they are much more palatable to the patient, I still stand by my favorite; and because of this preference of mine, you may probably judge me "an old fogy," and I will willingly abide the sentence, yielding myself freely to the penalty under the order of the court.

I will proceed now abruptly to narrate the cases in proof of the claim made in aborting this fierce disease:

Case No. 1. Mr. C., aged 18 years, of lymphatic temperament; came to my office suffering terribly with a carbuncle on the nape of his neck. The angry tumor was an inch in diameter, full of fever and very painful. It was three or four days old. The youthfulness of the patient made me afraid of a high grade of inflammatory action, and to hesitate about adopting the abortive treatment by one of the most powerful tonic medicines in the materia medica; and after much deliberation I resolved to compromise with my doubt by adopting for one day longer the old method of management. The tumor was then painted over with tincture of iodine, a linseed meal poultice well carbolized, and a dose of compound cathartic pills administered, to be followed, if necessary, on the following morning by 30 grains of hyposulphite of soda in half a tumblerful of water, admonishing him to report again next morning, promising myself, if there was no improvement, I would scarify freely and deeply into the swollen part to relieve the congestion of the blood vessels, and in this way ameliorate the tension of the irritated nerves. But, instead of being better next morning, it was decidedly worse. The tumescence had enlarged to two inches in diameter, was angrier in appearance and much more painful to the patient. I was mad at my dilatoriness in putting my theory to the test; the patient was mad because he received no relief, and the carbuncle seemed to be as angry as both of us put together. It was then that I recovered the courage of my convictions, and at once determined, come what might, to give my theory the chance of its life. The formula for the Kempe tonic was given him; the tumor was sponged with carbolized warm water, and a poultice of powdered cinchona bark wetted with brandy—to be remoistened every six hours—was applied to the tumor, with instructions to take the dose of hyposulphite of soda every night at bed time. He was requested to report again next morning, but he failed to do so. Nor did he return any more for four days; and, in the meantime I was on tenter hooks of agonizing suspense, troubled in mind as to the cause of the delay, and fearful that "the other fellow" had gotten the case in charge, and was, perhaps, at that moment scoring me with criticisms, and charging me with absolute ignorance of the nature and treatment of the disease. All during those days my feelings were wrought up to a pitch of painful anxiety. I even regretted ever seeing the patient, and over and over again it recurred to me that I had acted the part of redoubled fool for allowing the theory, however plausible it might be, to harken to the musical vibrations of the siren which captivated my judgment. However, he came at last, and with the sunshine on his handsome face, the storm passed away forever. His first remarks were, "Well, Doctor, I am well again." It will never be difficult to me to realize the great throbs of heartfelt relief and exultation which filled my happy brain at that moment. One glance at the place where the tumor had been assured me that he was correct. There was not a sign of it to be seen. He had simply remained away because he felt it not essential for his return. For an hour or so my exalted feelings remained stationary, but of a sudden, like a black cloud, an imp of doubt in the form of coincidence spread a deep gloom over my mind like a reaction, and again I was in despair. See how easily we are swayed by the waves of emotion. There was no comfort for me only in the hope of soon having other cases with which to prove my theory, or to abandon it altogether. In the meantime I worried myself no little by going over all the points of my diagnosis in the case. The end of it was a conclusive belief of its correctness. The image of the carbuncle was the same I had witnessed in quite a number of cases previously. There was no mistake at all.

Case No. 2 was a feeble white woman 60 years of age, who had for many days suffered from a carbuncle, three inches in diameter, on the dorsal region. It was well honey-combed, quaggy, and a thin pus was oozing from several points on its surface. This case, while it was in all other respects favorable for my tonic treatment, was a little forbidding by its duration being so near the sloughing period. It occurred to me, however, that its being almost the opposite of case No. 1, would, if cured by the tonic, go very far in sustaining my theory. The lady was therefore encouraged to hope for prompt relief, and put upon the same treatment of Mr. C.; that is, the hyposulphite of soda as a gentle aperient daily administered; a poultice of pulverized cinchona bark wetted with brandy; an occasional whisky toddy, and a dose of Kumpe tonic three times a day, after nourishing meals. My reason for prescribing the tonic immediately after meals was derived from the fact that many of my patients were distressed with nausea and vomiting when it was taken on an emply stomach, owing to its intense bitterness. This patient was under my care for only four or five days, and was free from suffering almost from the start, and dismissed me at last, very grateful for my services.

Case No. 3 was a stout-framed negro man, about 35 years of age. The carbuncle, two inches in diameter, was situated on the right scapular region, of four days' standing. He complained greatly, saying the pain was so excruciating that he got no sleep, night or day. The treatment did not differ in anything from that of cases Nos. 1 and 2. He was ordered to report every morning, but failed to do so. In fact, I saw him no more for ten days, and then accidentally met him on the road, and when I began scolding, he headed me off, saying "Dat big bile done got well right away."

It is upon these three cases that I base my claim of having, by a tonic treatment, succeeded in aborting carbuncle in mid career, as it were; in displacing its recognition from the surgical department to the medical repertory; and finally, to claim priority in introducing the rational treatment of the disease. It is quite likely that, if I had been so disposed, this flash of light might have by patent right become a source of revenue to my pocket, but like the several others that have illuminated my path, never a financial idea occurred to my mind, because, under the old regime, we were taught that it was our glorious mission to confer upon suffering humanity all the benefits in our power to bestow.

Let me close by repeating the fact that tonic medicines in skin diseases deserve much more interest by the profession than they have heretofore received.

